# Acute Posterior Multifocal Placoid Pigment Epitheliopathy as the Initial Manifestation of Sarcoidosis

**Published:** 2011-10

**Authors:** Adil Darugar, Alexis Mathian, Phuc LeHoang, Bahram Bodaghi

**Affiliations:** 1 Department of Ophthalmology, Pitié-Salpêtrière Hospital, Paris, France; 2 Department of Internal Medicine, Pitié-Salpêtrière Hospital, Paris, France

**Keywords:** APMPPE, Sarcoidosis, Dyspnea, Indocyanine Green Angiography, OCT

## Abstract

**Purpose:**

To report an undiagnosed case of systemic sarcoidosis manifesting with bilateral acute posterior multifocal placoid pigment epitheliopathy (APMPPE).

**Case Report:**

A 26-year-old Caucasian man was referred for management of unilateral visual loss together with a paracentral scotoma developing 2 weeks after a flu-like syndrome. Clinical signs and ancillary diagnostic investigations suggested APMPPE. Laboratory tests demonstrated elevated serum angiotensin converting enzyme and lysozyme levels. Chest CT-scan disclosed moderate hilar lymph node calcifications but QuantiFERON-TB gold test was negative and bronchoalveolar lavage and biopsies were unremarkable. Accessory salivary gland biopsy disclosed epithelioid and gigantocellular granuloma formation without caseum, confirming a diagnosis of sarcoidosis. The fellow eye was involved a few days later and the patient complained of dyspnea. Echocardiography disclosed severe granulomatous myocardial infiltration and high dose corticosteroids and intravenous cyclophosphamide were initiated. Systemic treatment controlled both cardiac and ocular lesions, and was tapered accordingly.

**Conclusion:**

The constellation of “white dot syndromes” and systemic symptoms necessitates a general work-up to exclude granulomatous disorders such as sarcoidosis or tuberculosis. Delayed diagnosis of cardiac sarcoidosis may have life-threatening consequences and the ophthalmologist may be the first physician to diagnose the condition.

## INTRODUCTION

In 1968, Gass described 3 young patients presenting with rapid central visual loss secondary to multifocal, yellow-white, placoid lesions at the level of the retinal pigment epithelium (RPE) and choroid. The disorder was transient and presumed to involve the RPE. It was named under the descriptive term of acute posterior multifocal placoid pigment epitheliopathy (APMPPE).^1^

The pathophysiology and the level of posterior segment involvement in APMPPE were controversial before the availability of indocyanine green angiography (ICGA) and optical coherence tomography (OCT). APMPPE is an uncommon condition presenting in otherwise healthy young adults. It may rarely be associated with a wide range of disorders such as tuberculosis,^2^ Wegener’s granulomatosis,^3^ cerebral vasculitis,^4^ vaccination for hepatitis B virus,^5^ adenovirus type 5 infection,^6^ Lyme disease^7^,^8^ and varicella vaccination^9^.

Herein, we present a young patient presenting with APMPPE as the initial manifestation of severe systemic sarcoidosis.

## CASE REPORT

A 26-year-old man was referred to our clinic for blurred vision and black spots in his left eye starting 3 days prior to referral. He was a healthy man with a history of allergic rhinitis and contact with cats. He reported a flu-like syndrome 2 weeks before the onset of visual symptoms which had regressed spontaneously. There were no signs of CNS involvement.

**Figure 1:**
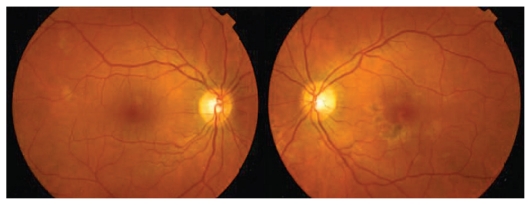
Fundus photograph at presentation reveals multifocal white-yellow lesions predominantly in the left eye.

At presentation, visual acuity was 20/20, the anterior segment was unremarkable, intraocular pressure (IOP) was normal, and the vitreous cavity was clear in both eyes. Multiple deep gray-white placoid lesions in the posterior pole of both eyes were observed on fundus examination (Fig. 1), some of which were partially pigmented.

**Figure 2:**
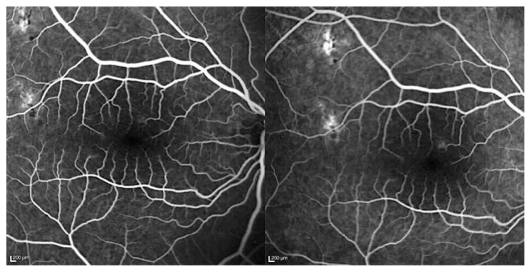
(a) Early and (b) intermediate fluorescein angiography frames show hyperfluorescence with moderate staining in the right eye.

**Figure 3:**
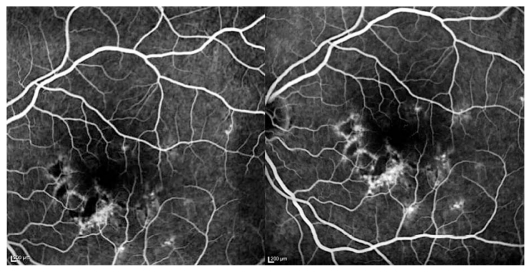
(a) Early and (b) intermediate fluorescein angiography frames show hyperfluorescence with moderate staining in the left eye.

**Figure 4:**
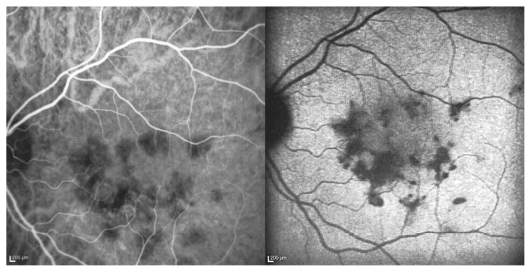
(a) Early and (b) late ICGA frames reveal more extensive areas of hypofluorescence in the left eye as compared to lesions observed on the fundus photograph.

**Figure 5:**
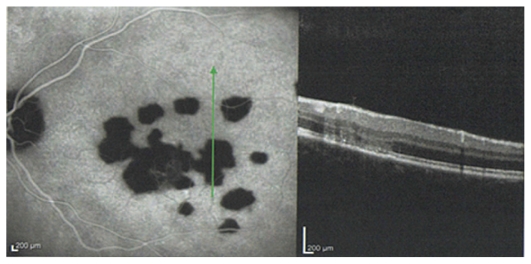
Spectral domain OCT shows hyper-reflectivity at the level of the RPE.

Goldmann visual field revealed multiple small paracentral scotoma in both eyes sparing the fovea. Fluorescein angiography disclosed early hyperfluorescent lesions at the posterior pole with late staining, without macular edema or papillitis (Figures 2 and 3). This appearance was compatible with old lesions together with mild active inflammation. ICGA demonstrated multiple hypofluorescent lesions during the whole sequence disclosing more lesions than apparent on fundus examination and fluorescein angiography (Fig. 4). OCT showed hyper-reflectivity and alterations at the level of the photoreceptors and RPE (Fig. 5). The multifocal electroretinogram was normal.

Ancillary tests revealed elevated serum levels of angiotensin converting enzyme (1.5 times normal) and lysozyme (1.8 times normal). Complete blood cell count, C-reactive protein, liver and renal function tests were normal. Tuberculin skin test was positive with an induration of 10 mm, however the patient had previous BCG (bacille Calmette-Guerin) vaccination and the QuantiFERON-TB Gold test was negative. Furthermore, Syphilis, Lyme and HIV serology, antinuclear antibody (ANA) and anti-neutrophil cytoplasmic antibody (ANCA) were all negative. Chest CT scan showed moderate hilar lymph node calcifications without adenomegaly and abdominal CT scan was normal.

Bronchoalveolar lavage disclosed an inflammatory mucosa and macrophage alveolitis, compatible with smoking. Bronchial biopsies were normal. Accessory salivary gland biopsy disclosed an epithelioid and gigantocellular granulomatous reaction without caseum confirming the diagnosis of sarcoidosis.

Twelve days after referral, the patient presented with the same symptoms in the fellow eye. Visual acuity was 20/20 in both eyes, and the anterior segments and IOP were also normal. Mild bilateral vitritis was noted and fundus examination revealed stable lesions which were more pigmented on the left side. Visual field had improved in the left eye and was normal in the right eye.

The patient was monitored every 2 weeks. Symptoms were decreasing and visual field defects were improving but the patient still complained of visual discomfort. Echocardiography disclosed severe cardiac involvement explaining his dyspnea. Based on cardiac alterations and after multidisplinary consultation, cyclophosphamide was initiated together with high dose systemic corticosteroids followed by slow tapering after stabilization. Visual discomfort decreased and visual acuity remained stable within two years of follow- up, with no further ocular relapses.

## DISCUSSION

After the initial report by Gass in the late 1960s, Ryan and Maumenee described nine other patients with APMPPE in 1972 and made similar observations favoring Gass’s hypothesis that the RPE is primarily involved rather than the choroid.^10^ However, Deutman et al later proposed that acute inflammation at the level of the choriocapillaris is the underlying pathophysiology in APMPPE and that RPE changes are a subsequent manifestation.^11^ Choroidal hypoperfusion (established by ICGA) could be a likely mechanism of injury leading to RPE scarring without permanent retinal photoreceptor damage.^12^ This can explain why the visual prognosis is usually, but not invariably, good.

Spectral domain OCT in the acute phase of APMMPE shows disruption of the hyper- reflective bands representing the inner-outer segment junction and the Verhoeff membrane. However, these lesions are also seen after healing in areas that initially appeared normal on OCT. It is hypothesized that in addition to the inflammatory component, secondary degenerative changes of the photoreceptors and RPE may play a role in tissue destruction, resulting in the commonly recognized pigmentary changes observed in resolved APMPPE.^13^

Sarcoidosis affects the eye and optic nerve in different manners and approximately 20% of patients have already developed ocular lesions at the time of initial examination. In biopsy-proven disease the incidence seems even higher.^14^ Anterior segment disease occurs more commonly, usually manifesting as chronic granulomatous uveitis. Posterior segment involvement occurs less frequently and is rarely isolated. Chorioretinitis, periphlebitis, and chorioretinal nodules are the most frequently described lesions.^15^–^16^ RPE alterations usually occur peripherally and are not associated with visual disturbances.^16^ Vitreous opacities and choroidal granulomas may also occur. Conjunctival and lacrimal gland involvement, papilledema, and direct granulomatous involvement of the optic nerve^17^ and chiasma^18^ have also been reported.

Dick et al were the first to report the association between APMPPE and sarcoidosis.^19^ They described a 24-year-old male patient presenting with a flu-like prodrome followed by development of multiple scotomata 10 days later. After another 6 weeks, he presented with bilateral anterior uveitis and small recurrent oral ulcers. The patient demonstrated CNS involvement with myeloradiculopathy and polyadenopathy with epithelioid granulomas, giant cells and occasional central necrosis. Renal biopsy confirmed the diagnosis of sarcoidosis by demonstrating non-caseating granuloma. Symptoms resolved with high dose oral prednisolone.

The cause of sarcoidosis remains unknown and its diagnosis can be challenging. Clinical presentation is multisystemic and can be confusing. Ancillary tests are helpful but none of them are specific and histological proof is not always available. CNS involvement is rare and represents about 5% of cases.^20^ Transient ischemic attacks and strokes occur very rarely, in contrast to several pathological descriptions of perivascular and vascular infiltration of meningeal and cerebral vessels by granuloma often with subclinical infarctions.^21^

Histopathological characteristics of APMPPE have previously been reported in a patient with CNS involvement. The choroid showed granulomatous inflammation with focal disruption of the RPE but the granulomas were not located in or near the vessels.^22^ It was hypothesized that, APMPPE results from ischemia of the choriocapillaris due to the narrowing of upstream medium arteries by granular infiltration.

In our patient, APMPPE was the first manifestation of sarcoidosis. Investigations revealed subclinical lung disease. There was no sign of central nervous system involvement. Many cases with APMPPE associated with cerebral vasculitis have been reported but only a few had histological confirmation.^4^,^23^–^26^ Most of the cases were controlled with steroids and/or immunosuppressive therapy; neurosarcoidosis may occur in these cases.

The frequent occurrence of a precedent flu-like syndrome raises the issue of a triggering infectious agent and a viral illness has been suspected. Adenovirus type 5 has been identified in 2 patients with APMPPE.^6^,^27^ Vaccination has also been reported in association with APMPPE.^5^,^9^ These events may be considered as the trigger for immune dysregulation leading to an inflammatory process.

Sarcoidosis is the major differential diagnosis in patients with APMPPE. A positive biopsy is the most appropriate way to confirm the diagnosis. Lyme disease and tuberculosis should also be ruled out. In our patient, certain signs including a positive tuberculin skin test and mediastinal lymph node calcifications were in favor of tuberculosis. However, the patient had previous BCG vaccination, the Quantiferon-TB Gold was negative, and mediastinal lesions were nonspecific. Moreover, high dose corticosteroids and cyclophosphamide did not lead to reactivation of presumed latent tuberculosis. Internists and neurologists played active roles in the management of this patient. In the absence of histological confirmation, other parameters should be considered in order to propose presumed, probable or possible sarcoidosis.^28^

Due to mild to moderate ocular involvement in our patient and the absence of significant complications, we did not start anti-inflammatory treatment, initially. However, cardiac involvement in patients with sarcoidosis has been reported in 25–39% of patients and is responsible for up to 85% of deaths attributed to the disease, often due to sudden cardiac death. Therefore, aggressive anti-inflammatory therapy and immunosuppressive drugs were initiated in our patient. The ophthalmologist may be the first physician to be confronted with the disease and plays a major role in establishing the diagnosis and referring the patient for appropriate therapy, thus avoiding a fatal outcome.
